# Supplementary material for: How Temperatures May Affect the Synthesis of Fatty Acids during Olive Fruit Ripening: Genes at Work in the Field
Source: Plants (Basel). 2022 Dec 22;12(1):54. doi: 10.3390/plants12010054 (PMC9824132; doi:10.3390/plants12010054)
Supplement: Supplementary file 1 [file plants-12-00054-s001.zip › plants-2082564-supplementary.pdf]

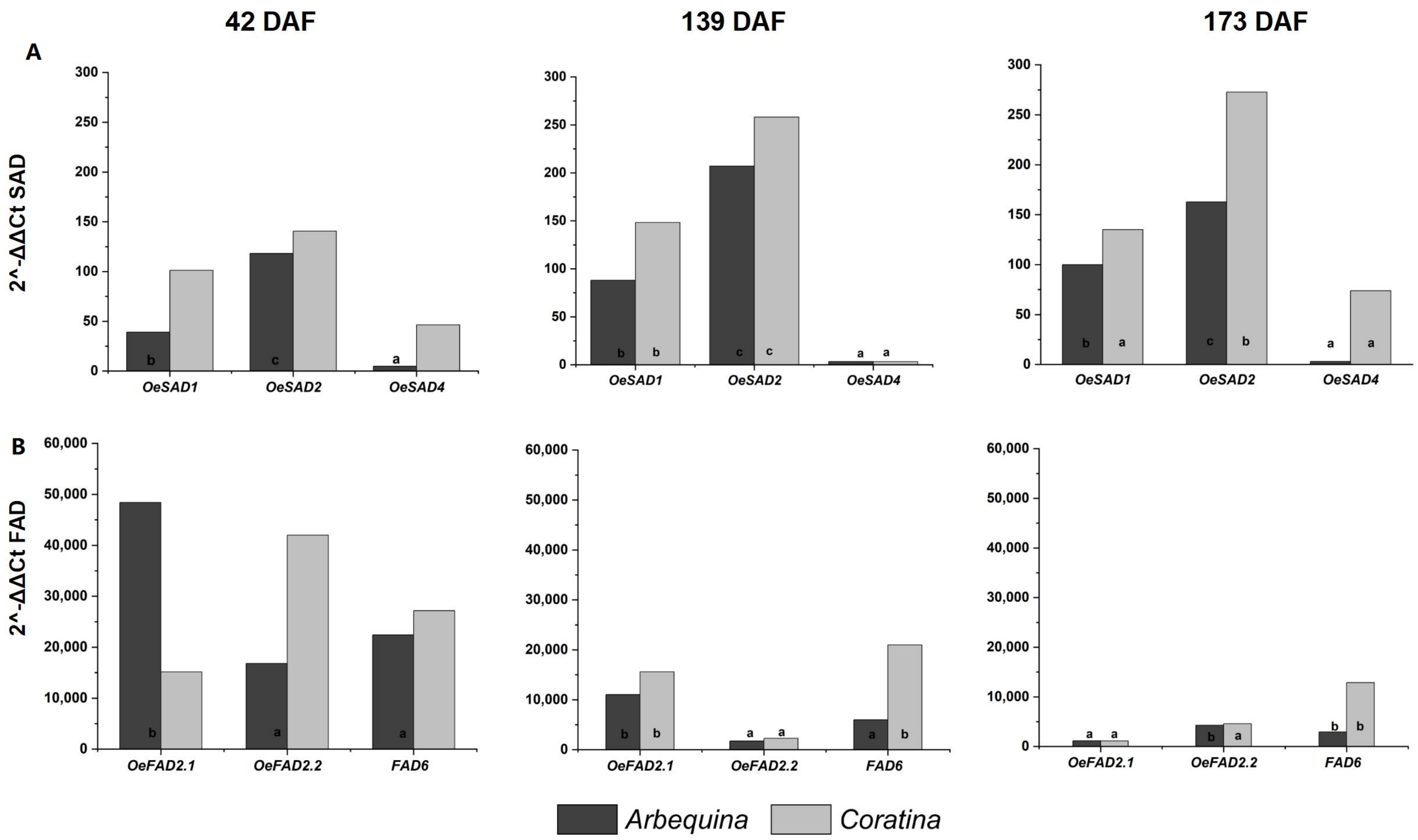

Supplementary

Figure S1 A and B. Expression *OeSAD* (A) *OeFAD* (B) and gene families at three fruit phenological stages (42, 139 and 173 days after full flowering (DAF)) in the cvs. Arbequina and Coratina considering the seven growing environments analyzed. Different letters correspond to significant differences at $p < 0.05$ among genes for a given phenological stage.
